# A phase III, 52‐week, open‐label study to evaluate the safety and efficacy of 5% sofpironium bromide (BBI‐4000) gel in Japanese patients with primary axillary hyperhidrosis

**DOI:** 10.1111/1346-8138.15927

**Published:** 2021-05-26

**Authors:** Tomoko Fujimoto, Yoichiro Abe, Masaru Igarashi, Akiko Ishikoh, Tokuya Omi, Hiroki Kanda, Hiroto Kitahara, Miwako Kinoshita, Ichiro Nakasu, Naoko Hattori, Yuki Horiuchi, Ryuji Maruyama, Haruko Mizutani, Yoshiyuki Murakami, Chiharu Watanabe, Akihiro Kume, Takaaki Hanafusa, Masamitsu Hamaguchi, Akira Yoshioka, Yuriko Egami, Keizo Matsuo, Tomoko Matsuda, Motoki Akamatsu, Toshiyuki Yorozuya, Shinichi Takayama, Hiroo Yokozeki

**Affiliations:** ^1^ Ikebukuro Nishiguchi Fukurou Dermatology Clinic Tokyo Japan; ^2^ Department of Pain Clinic NTT Medical Center Tokyo Tokyo Japan; ^3^ Igarashi Dermatology Clinic Tokyo Japan; ^4^ Kaminoge Hifuka Clinic Tokyo Japan; ^5^ Department of Dermatology Queen's Square Medical Center Kanagawa Japan; ^6^ Mita Dermatology Clinic Tokyo Japan; ^7^ Kitahara Dermatology Clinic Tokyo Japan; ^8^ Kinoshita Dermatology Clinic Tokyo Japan; ^9^ Nemunoki Dermatology Clinic Kanagawa Japan; ^10^ Naoko Dermatology Clinic Tokyo Japan; ^11^ Akihabara Skin Clinic Tokyo Japan; ^12^ Maruyama Dermatology Clinic Tokyo Japan; ^13^ Mizutani Dermatology Clinic Tokyo Japan; ^14^ Mildix Skin Clinic Tokyo Japan; ^15^ Chiharu Dermatology Clinic Saitama Japan; ^16^ Dermatology and Ophthalmology Kume Clinic Osaka Japan; ^17^ Senri‐Chuo Hanafusa Dermatology Clinic Osaka Japan; ^18^ Hamaguchi Clinic Osaka Japan; ^19^ Yoshioka Dermatology Clinic Osaka Japan; ^20^ Ekihigashi Dermatology and Allergology Clinic Fukuoka Japan; ^21^ Matsuo Clinic Fukuoka Japan; ^22^ Tomoko Matsuda Dermatological Clinic Fukuoka Japan; ^23^ Kaken Pharmaceutical Co., Ltd. Tokyo Japan; ^24^ Department of Dermatology Tokyo Medical and Dental University Tokyo Japan

**Keywords:** 52‐week study, BBI‐4000, phase III study, primary axillary hyperhidrosis, sofpironium bromide gel

## Abstract

A long‐term study was conducted in Japanese patients with primary axillary hyperhidrosis who completed the preceding 6‐week phase III, confirmatory study of 5% sofpironium bromide gel (hereinafter referred to as sofpironium) to evaluate the safety and efficacy of 52‐week treatment with sofpironium. In the long‐term study, 185 patients who completed the confirmatory study (94 and 91 patients in the vehicle and sofpironium groups, respectively) started to receive sofpironium (switching and extension groups, respectively), and all these patients were included in both the full analysis set (FAS) and the safety analysis set (SAF). In the FAS, there were more females than males (73.0% vs. 27.0%), and median age was 38.0 years. A total of 161 patients (86 and 75 patients in the switching and extension groups, respectively) completed the study at week 52. The proportions of patients with hyperhidrosis disease severity score of 1 or 2 and a 50% or more reduction in total gravimetric weight of sweat were 57.4% in the switching group and 58.2% in the extension group at week 52. The proportions of patients who achieved this efficacy end‐point in the long‐term study were similar to that (53.9%) in the sofpironium group in the confirmatory study. In the SAF, the incidences of adverse events (AEs) were 80.9% in the switching group and 83.5% in the extension group, and the incidences of adverse drug reactions were 39.4% and 45.1%, respectively. AEs that occurred in at least 20% of patients in both treatment groups were application site dermatitis (25.5% and 33.0%, respectively) and nasopharyngitis (31.9% and 23.1%, respectively). Reported AEs were generally mild, and there were no deaths. Serious AEs occurred in three patients, but none were considered related to the study drug. In this study, the efficacy of sofpironium was maintained during 52‐week treatment, and no new safety risk was observed.

## 1 INTRODUCTION

Hyperhidrosis, a skin disease involving excessive sweating beyond what is physiologically required for thermoregulation, is clinically diagnosed when excessive sweating results in emotional, physical, or social distress and impairs patients’ quality of life (QOL). Hyperhidrosis can be primary or secondary. Approximately 93% of cases of hyperhidrosis are primary, and more than 90% of cases of primary hyperhidrosis are focal hyperhidrosis affecting the axillae, palms, soles, and craniofacial areas.^1^ According to an epidemiological study conducted in Japan from 2009 to 2010 (5807 respondents), the prevalence of primary axillary hyperhidrosis was 5.75% (334 persons), with a mean onset age of 19.5 years.^2^

Primary axillary hyperhidrosis is a refractory disease that not only limits daily and social activities, but also causes psychological or emotional distress to the patients.^3^, ^4^, ^5^ In addition, this disease interferes with daily activities of the patients because of need for frequent changes of clothes or showers, with limited choice of clothing.^6^ Primary axillary hyperhidrosis is also characterized by a very high prevalence in the socially active and productive generation.^3^ This disease often manifests in puberty and adolescence and persists for the rest of life,^7^ requiring long‐term treatment unless curatively treated with, for instance, surgical therapy.

Sofpironium bromide (BBI‐4000) is a muscarinic acetylcholine, M3 receptor ligand that resembles anticholinergic glycopyrronium bromide in chemical structure and has been developed as a retrometabolically designed drug with ethyl ester residue.^8^, ^9^ It is considered that sofpironium bromide has a high binding affinity for the M3 acetylcholinergic receptor at the local site of administration, but is hydrolyzed at the ester linkage to less active metabolites upon entry into blood. Therefore, sofpironium bromide is expected to be effective in reducing sweating in primary axillary hyperhidrosis and to be associated with fewer systemic anticholinergic adverse drug reactions (ADRs) compared to the parent drug, glycopyrronium.

We previously conducted a phase III study to verify the efficacy and safety of 5% sofpironium bromide gel (hereinafter referred to as sofpironium) administrated for 6 weeks in Japanese patients with primary axillary hyperhidrosis, and demonstrated the efficacy and safety of sofpironium.^10^ In September 2020, sofpironium (ECCLOCK^®^ gel 5%; Kaken Pharmaceutical Co., Ltd., Japan) was approved in Japan for the topical treatment of primary axillary hyperhidrosis. However, sofpironium had not been administrated for more than 6 weeks, and the safety and efficacy of long‐term treatment of sofpironium had not been studied. According to the ICH‐E1 guideline, the safety of drugs intended for long‐term treatment of non‐life‐threatening conditions must be assessed based on the results of a 12‐month long‐term safety study with relevant investigational products. Therefore, we conducted a long‐term study of sofpironium in Japanese patients with primary axillary hyperhidrosis who completed the preceding 6‐week phase III study (confirmatory study) to evaluate the safety and efficacy of sofpironium administrated once daily for 52 weeks.

## 2 METHODS

### 2.1 Study design

This was an extended study following the previous confirmatory study and designed as a multi‐center, open‐label, uncontrolled study. The duration of treatment was 52 weeks after entry from the confirmatory study. Allowing for dropouts during the study, the target sample size was determined to be 150 patients, in accordance with the ICH‐E1 guideline. This study was started after the protocol was approved by the institutional review board (IRB). The study was conducted in compliance with the protocol approved by the IRB, the principles of the Declaration of Helsinki, and the ministerial ordinance concerning Good Clinical Practice (GCP) (Ordinance of the Ministry of Health and Welfare No. 28, 1997) and its revisions. Written informed consent was obtained from all patients before participation in the study. The study period was from 28 June 2018 to 5 November 2019 (date of final observation) (JAPIC no. JapicCTI‐184003).

### 2.2 Study patients

Japanese patients with primary axillary hyperhidrosis who participated in the confirmatory study^10^ and completed 6‐week treatment with a treatment compliance rate of 80% or more were included in this study.

### 2.3 Treatment method

An adequate amount of sofpironium was thoroughly applied, namely one actuation of the product per axilla once daily at bedtime for 52 weeks. Application, not at bedtime, was allowed only once daily.

Concomitant medications/therapy used with sofpironium during the study, as listed below, were investigated. Patients who used relevant medication/therapy during a given period of time (time in parentheses) before week 52 after entry into the study were identified to evaluate the efficacy at week 52 in these subgroups.

1. Systemic and topical anticholinergics (within 30 days)
2. Oral cholinergic agonists, serotonin agonists, β‐blockers, α‐adrenergic agonists, dopamine partial agonists, and tricyclic antidepressants (within 30 days)
3. Aluminum chloride and medications for hyperhidrosis approved outside Japan (within 30 days)
4. Botulinum toxin (within 9 months)
5. Over‐the‐counter (OTC) pharmaceuticals, quasi‐drugs, or cosmetics containing astringents with anhidrotic effects (within 7 days)
6. Herbal medicines for reducing symptoms of hyperhidrosis (within 7 days)
7. Axillary laser therapy, surgical therapy, and thoracic sympathectomy (throughout the study)

### 2.4 End‐points

The efficacy end‐points were: (i) proportion of patients who satisfied both criteria with a Hyperhidrosis Disease Severity Score (HDSS)^11^ of 1 or 2 and a 50% or more reduction in total gravimetric weight of sweat; (ii) proportion of patients with a HDSS of 1 or 2; (iii) proportion of patients with a 50% or more reduction in total gravimetric weight of sweat; (iv) change in total gravimetric weight of sweat; (v) change in Dermatology Life Quality Index (DLQI) (for axillary hyperhidrosis) score;^12^ (vi) proportion of patients with an improvement of 1.5 or more in Hyperhidrosis Disease Severity Measure – Axillary (HDSM‐Ax) score;^13^ and (vii) change in HDSM‐Ax score. The gravimetric weight of sweat was measured as the difference in filter paper weight before and after 5 min of contact with the affected area (axilla). The HDSS^11^ is used to assess the severity of primary focal hyperhidrosis by classifying subjective symptoms as follows:

1. Sweating is never noticeable and never interferes with daily activities
2. Sweating is tolerable, but sometimes interferes with daily activities
3. Sweating is barely tolerable and frequently interferes with daily activities
4. Sweating is intolerable and always interferes with daily activities

The DLQI,^12^ which is designed to evaluate the skin disease‐related QOL, was modified into a DLQI for axillary hyperhidrosis to make it more suitable in assessment of primary axillary hyperhidrosis. Responses to 10 questions were scored and summed to obtain the DLQI score for axillary hyperhidrosis. The HDSM‐Ax^13^ is a severity scale for axillary hyperhidrosis, and in this study the score was evaluated as the mean of response scores to 11 questions from 1–3 of the HDSM‐Ax scale.

**FIGURE 1:**
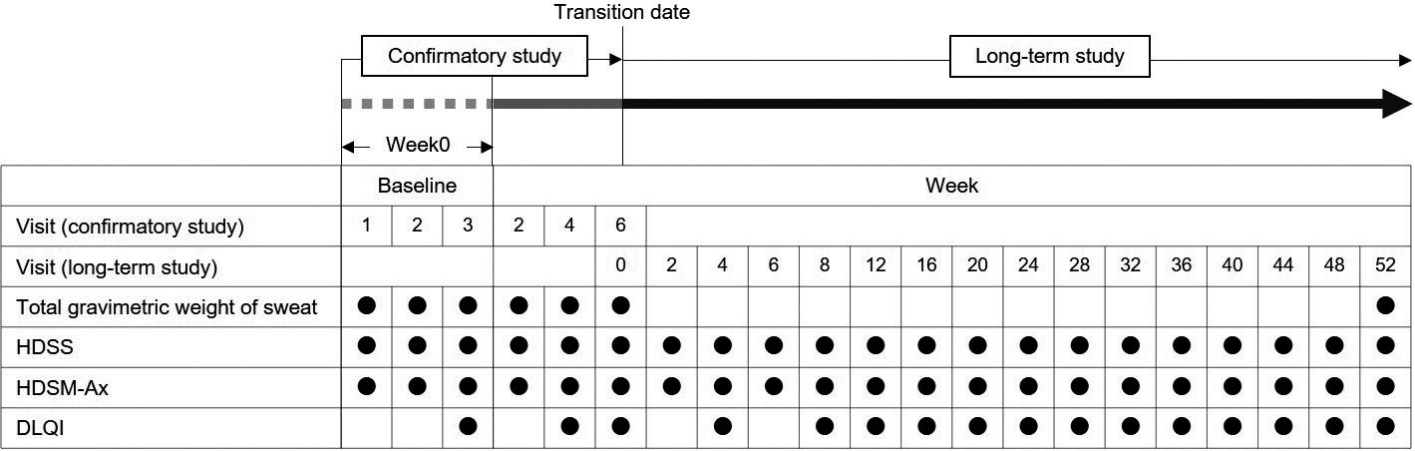
Timing of evaluation on the confirmatory study and long‐term study. Abbreviations: DLQI, dermatology life quality index; HDSM‐Ax, hyperhidrosis disease severity measure‐axillary; HDSS, hyperhidrosis disease severity score.

The schedule is presented in Figure 1. The HDSS, HDSM‐Ax score, and DLQI score were generally measured at each visit after entry into this study from the confirmatory study. Gravimetric weight of sweat was measured only at week 52 after entry from the confirmatory study.

The safety end‐points were adverse events (AEs), local tolerability (assessed by the physician and patients), vital signs, and laboratory values (hematology, biochemistry, and urinalysis). AEs were coded by systemic organ class (SOC) and preferred term (PT) according to the MedDRA/J version 21.1. An ADR was defined as an AE that was reasonably considered at least possibly related to the study drug and for which a causal relationship with the study drug could not be ruled out. An AE that, in the opinion of the sponsor with reference to that of the investigator, may be attributable to an anticholinergic effect was considered as an anticholinergic AE if the medical expert agreed. Multiple episodes of the same AE in the same patient were counted as one patient in calculation of the incidence. For local tolerability, the physician assessed dryness, erythema, and scaling on a 5‐point score (0: none, 1: minimal, 2: mild, 3: moderate, 4: severe) and patients assessed burning sensation and itching on a 5‐point score (0: none, 1: very mild, 2: mild, 3: moderate, 4: severe).

### 2.5 Analysis

For each end‐point, the mean, standard deviation (SD), median, minimum to maximum, proportion (%) of patients, and 95% confidence interval (CI) at each assessment time point were calculated for each treatment group. Values at baseline 3 in the confirmatory study^10^ were used as the baseline values in the present study. For the efficacy end‐points (iv), (v), and (vii), that is, changes in mean score, an observed case approach was used in which only observed values were analyzed, without imputation of missing values. For the efficacy end‐points (i), (ii), (iii), and (vi), that is, proportions of patients who met certain criteria, patients for whom data were missing were handled as patients who failed to meet the criteria. SAS^®^ version 9.4 software (SAS Institute Inc.) was used for data analysis.

## 3 RESULTS

### 3.1 Study patients

**FIGURE 2:**
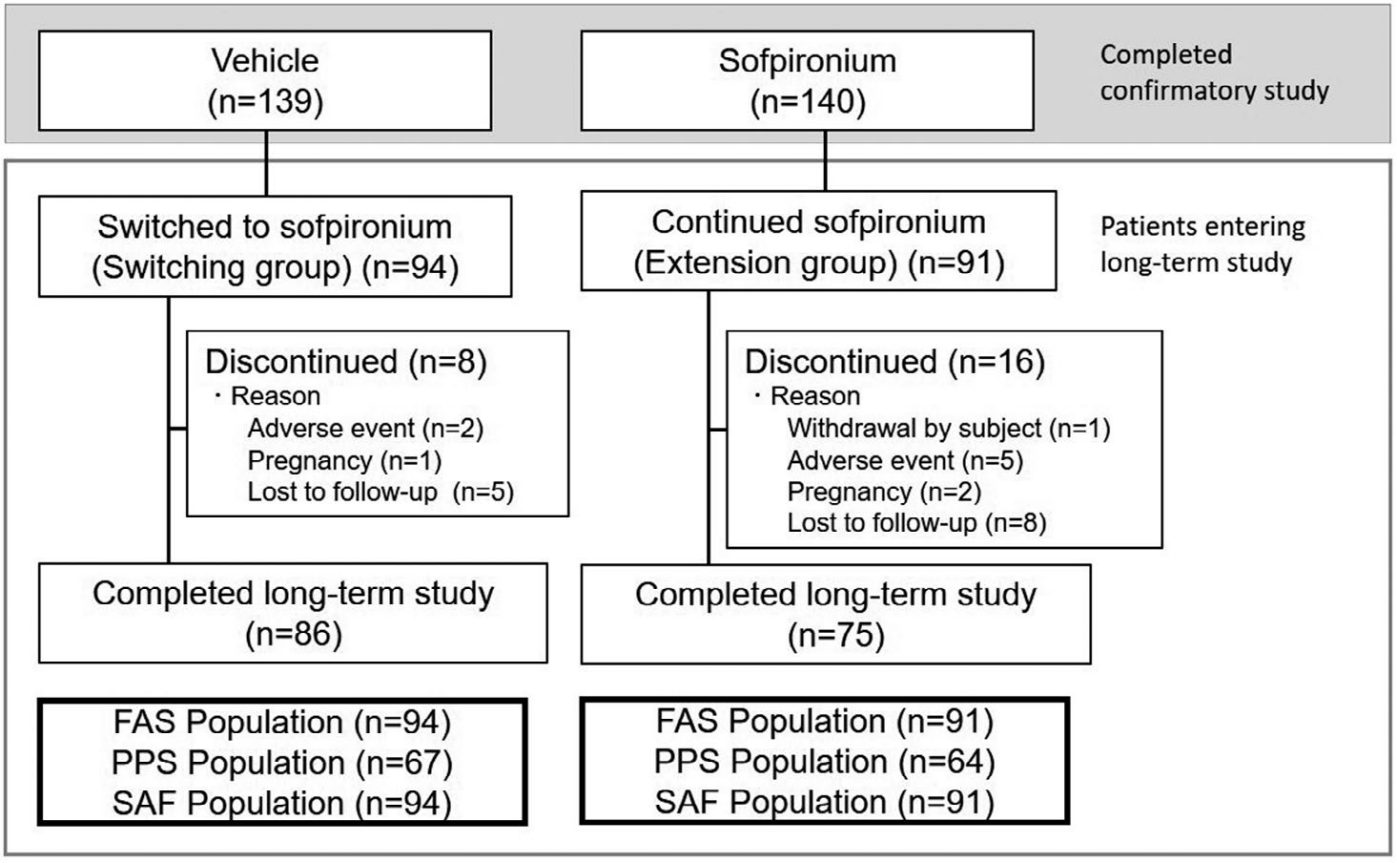
Patient disposition. Abbreviations: FAS, full analysis set; PPS, per protocol set; SAF, safety analysis set.

The disposition of study patients is presented in Figure 2. In the preceding 6‐week confirmatory study of sofpironium (multi‐center, randomized, double‐blind, vehicle‐controlled, parallel‐group study), 139 and 140 Japanese patients with primary axillary hyperhidrosis in the vehicle and sofpironium groups completed study treatment, respectively.^10^ Of these patients, 185 patients who provided written informed consent and met the eligibility criteria for the present long‐term treatment study (94 patients in the vehicle group and 91 patients in the sofpironium group in the 6‐week confirmatory study) started to receive sofpironium, and enrollment was then terminated during the registration period, because the target sample size (185 patients) was achieved. The population of patients who received the vehicle in the 6‐week confirmatory study and were then switched to sofpironium in the long‐term study was referred to as the switching group, and the population of patients who received sofpironium in the 6‐week confirmatory study and continued to receive sofpironium in the long‐term study was referred to as the extension group. Of the 185 patients (94 patients in the switching group and 91 patients in the extension group) who started to receive sofpironium, 161 patients (86 patients in the switching group and 75 patients in the extension group) completed the study at week 52 (Figure 2). Eight patients in the switching group and 16 patients in the extension group were discontinued from the study before week 52 due to AE, pregnancy, or failure to visit the study site in the switching group, and due to withdrawal of consent, AE, pregnancy, or failure to visit the study site in the extension group. All of the 185 patients enrolled in the long‐term study were included in the primary efficacy analysis set (full analysis set: FAS) and the safety analysis set (SAF).

### 3.2 Patient characteristics

Among the 185 patients in the FAS, there were more females than males (73.0% vs. 27.0%), and the median age was 38.0 years (Table 1). There were no imbalances in age, sex, or body mass index between the two groups.

**TABLE 1:** Baseline^a^ characteristics and sofpironium treatment status (FAS)

	Switching group (n = 94)	Extension group (n = 91)	Total (n = 185)
Age (years)			
Mean ± SD	38.2 ± 12.64	37.2 ± 14.21	37.7 ± 13.41
Median	39.0	36.0	38.0
Min–Max	13–72	14–72	13–72
Sex (%)			
Male	25 (26.6%)	25 (27.5%)	50 (27.0%)
Female	69 (73.4%)	66 (72.5%)	135 (73.0%)
BMI (kg/m^2^)			
Mean ± SD	21.88 ± 3.142	21.82 ± 3.412	21.85 ± 3.269
Median	21.12	21.37	21.16
Min–Max	17.3–32.4	14.8–34.7	14.8–34.7
Total gravimetric weight of sweat (mg)			
Mean ± SD	220.4 ± 166.08	176.9 ± 148.20	199.0 ± 158.62
Median	185.5	145.0	152.0
Min–Max	0–778	3–837	0–837
HDSS, n (%)			
Grade 3	62 (66.0%)	54 (59.3%)	116 (62.7%)
Grade 4	32 (34.0%)	37 (40.7%)	69 (37.3%)
HDSM‐Ax score			
Mean ± SD	3.10 ± 0.545	3.09 ± 0.532	3.09 ± 0.538
Median	3.09	3.09	3.09
Min‐Max	2.0–4.0	2.0–4.0	2.0–4.0
DLQI total score			
Mean ± SD	11.1 ± 4.47	12.0 ± 5.27	11.5 ± 4.88
Median	11.0	11.0	11.0
Min–Max	0–22	2–26	0–26
Timing of treatment, n (%)			
Always applied at bedtime	48 (51.1)	36 (39.6)	84 (45.4)
Constantly applied at a time other than bedtime	1 (1.1)	1 (1.1)	2 (1.1)
Temporarily applied at a time other than bedtime	45 (47.9)	54 (59.3)	99 (53.5)
Concomitant medication/therapy, n (%)			
Systemic or topical anticholinergics	2 (2.1)	1 (1.1)	3 (1.6)
Oral cholinergic agonists, serotonin agonists, β‐blockers, α‐adrenergic agonists, dopamine partial agonists, or tricyclic antidepressants	0	1 (1.1)	1 (0.5)
Aluminum chloride or medications for hyperhidrosis approved outside Japan	3 (3.2)	2 (2.2)	5 (2.7)
Botulinum toxin (axillary administration)	1 (1.1)	3 (3.3)	4 (2.2)
Over‐the‐counter pharmaceuticals, quasi‐drugs or cosmetics containing astringents with anhidrotic effects	3 (3.2)	2 (2.2)	5 (2.7)
Axillary laser therapy, surgical therapy, or thoracic sympathectomy	1 (1.1)	0	1 (0.5)

^a^ Baseline is defined as the value at baseline 3 in previous confirmatory study.

Abbreviations: BMI, body mass index; DLQI, dermatology life quality index; FAS, full analysis set; HDSM‐Ax, hyperhidrosis disease severity measure‐axillary; HDSS, hyperhidrosis disease severity score; Max, maximum; Min, minimum; SD, standard deviation.

### 3.3 Sofpironium treatment status

The median duration of sofpironium treatment (from initial treatment day + 1 day to final treatment day) was 361 days and usage rate of study treatment in individual patients were 100.0% in all periods. Of the 185 patients in the FAS, 84 patients (45.4%) applied all doses of sofpironium at bedtime. The remaining 101 patients (54.6%) applied at least one dose of sofpironium at a time other than bedtime (Table 1), and the most common reason for that was forgetting to take medication in 91 patients (49.2%), followed by inconvenient scheduling in 37 patients (20.0%), and poor health condition in 22 patients (11.9%).

### 3.4 Concomitant medication/therapy status

When the evaluation at week 52 might be affected (see the Methods section), three patients (1.6%) received systemic or topical anticholinergics concomitantly, one patient (0.5%) received oral cholinergic agonists, serotonin agonists, β‐blockers, α‐adrenergic agonists, dopamine partial agonists, or tricyclic antidepressants, five patients (2.7%) received aluminum chloride or medications for hyperhidrosis approved outside Japan, four patients (2.2%) received botulinum toxin (axillary administration), five patients (2.7%) received styptic‐containing, antiperspirant OTC medications, quasi‐pharmaceutical products, or cosmetics, and one patient (0.5%) received axillary laser therapy, surgical therapy, or thoracic sympathectomy (Table 1).

### 3.5 Efficacy (FAS)

#### 3.5.1 Efficacy end‐points

##### (i) Proportion of patients with a HDSS of 1 or 2 and a 50% or more reduction in total gravimetric weight of sweat

The proportion of patients with a HDSS of 1 or 2 at week 52 and a 50% or more reduction in total gravimetric weight of sweat from baseline to week 52 was 57.4% in the switching group and 58.2% in the extension group (Table 2).

**TABLE 2:** Summary of efficacy end‐points at week 52 (FAS)

Efficacy end‐points	Switching group (n = 94)	Extension group (n = 91)
Patients with a HDSS of 1 or 2, and a ≥50% reduction in total gravimetric weight of sweat from baseline^a^ to week 52, n (%)	54 (57.4%)	53 (58.2%)
Patients with a HDSS of 1 or 2 at week 52, n (%)	72 (76.6%)	65 (71.4%)
Patients with a ≥50% reduction in total gravimetric weight of sweat from baseline^a^ at week 52, n (%)	62 (66.0%)	61 (67.0%)
Change in total gravimetric weight of sweat from baseline^a^ to week 52, mean ± SD	−157.7 ± 178.08 mg	−141.6 ± 168.47 mg
Change in DLQI total score from baseline^a^ to week 52, mean ± SD	−8.8 ± 4.65	−9.7 ± 5.08
Patients with improvement ≥1.5 in HDSM‐Ax score from baseline^a^ to week 52, n (%)	65 (69.1%)	57 (62.6%)
Change in HDSM‐Ax score from baseline^a^ to week 52, mean ± SD	−2.13 ± 0.852	−2.07 ± 0.833

Note

For the efficacy end‐points, that is, proportions of patients who met certain criteria, patients for whom data were missing were handled as patients who failed to meet the criteria.

^a^ Baseline is defined as the value at baseline 3 in previous confirmatory study.

Abbreviations: DLQI, dermatology life quality index; FAS, full analysis set; HDSM‐Ax, hyperhidrosis disease severity measure‐axillary; HDSS, hyperhidrosis disease severity score; SD, standard deviation.

##### (ii) Proportion of patients with a HDSS of 1 or 2

**FIGURE 3:**
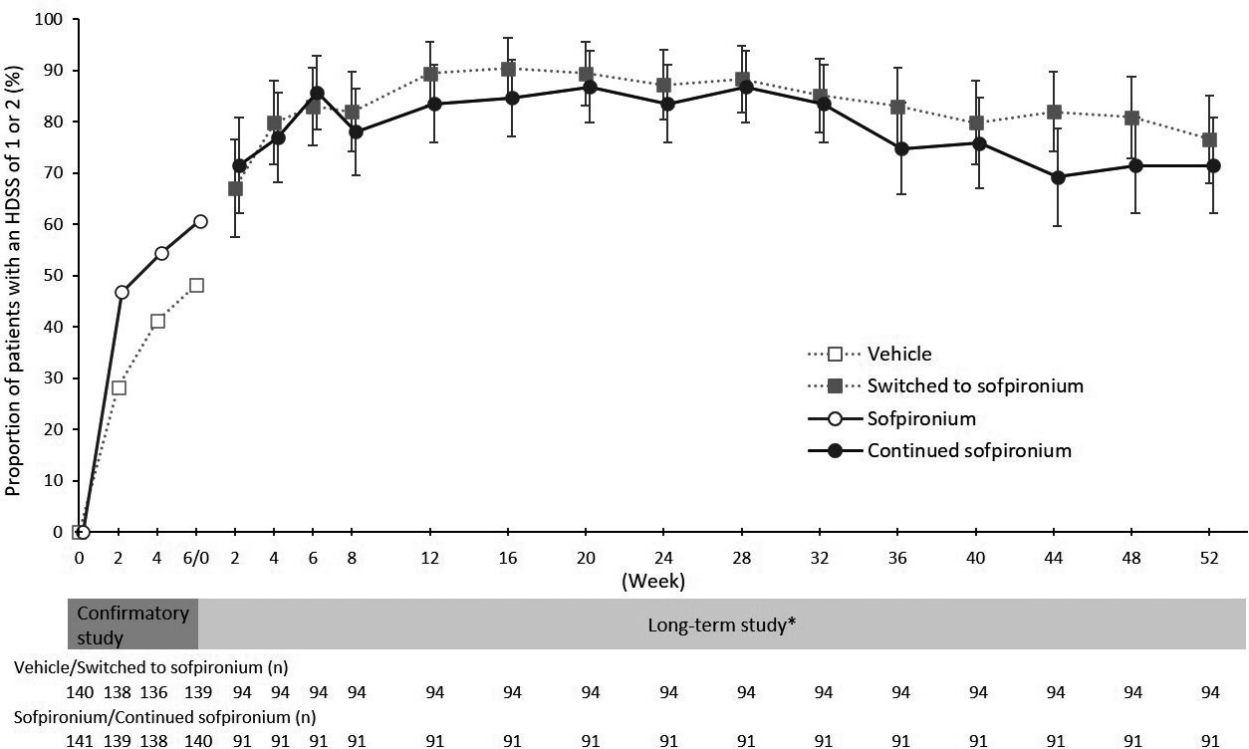
Change in proportion of patients with a Hyperhidrosis Disease Severity Score (HDSS) of 1 or 2. *Baseline is defined as the value at baseline 3 in the confirmatory study. Error bar shows 95% confidence interval. The patients for whom data were missing were handled as patients who failed to meet the criteria

The proportion of patients with a HDSS of 1 or 2 was 67.0% (63/94 patients) in the switching group and 71.4% (65/91 patients) in the extension group at week 2, 83.0% (78/94 patients) in the switching group and 85.7% (78/91 patients) in the extension group at week 6, 87.2% (82/94 patients) in the switching group and 83.5% (76/91 patients) in the extension group at week 24, and 76.6% (72/94 patients) in the switching group and 71.4% (65/91 patients) in the extension group at week 52 (Table 2 and Figure 3). The proportion of patients increased from the baseline of the long‐term study to week 6, and remained almost the same thereafter. In the aforementioned analysis in the FAS, patients for whom data were missing due to, for instance, withdrawal from the study were handled as patients who failed to meet the criteria (HDSS was not 1 or 2). When calculated post‐hoc using an observed case approach, the proportion of patients with a HDSS of 1 or 2 at week 52 was 84.7% (72/85 patients) in the switching group and 86.7% (65/75 patients) in the extension group.

##### (iii) Proportion of patients with a 50% or more reduction in total gravimetric weight of sweat

The proportion of patients with a 50% or more reduction in total gravimetric weight of sweat from baseline to week 52 was 66.0% (62/94 patients) in the switching group and 67.0% (61/91 patients) in the extension group (Table 2).

##### (iv) Change in total gravimetric weight of sweat

The change (mean ± SD) in total gravimetric weight of sweat from baseline to week 52 was −157.7 ± 178.08 mg in the switching group and −141.6 ± 168.47 mg in the extension group (Table 2).

##### (v) Change in DLQI score

**FIGURE 4:**
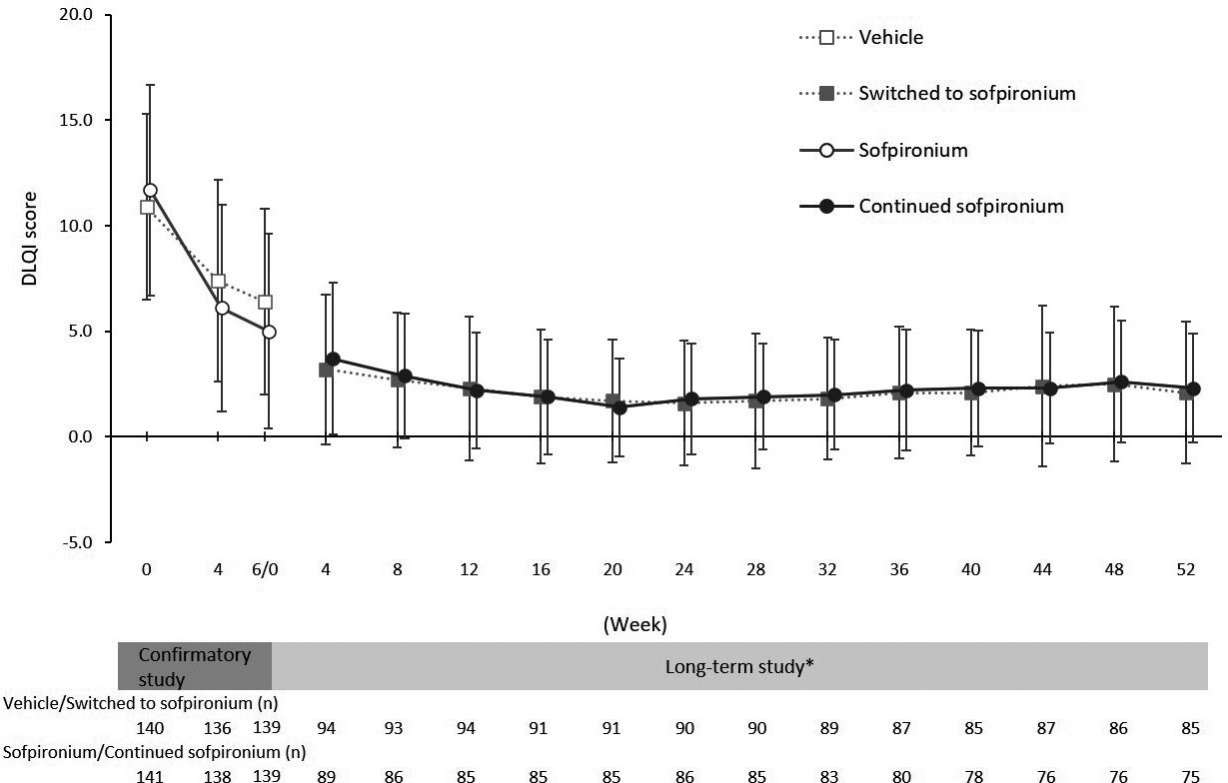
Change in Dermatology Life Quality Index (DLQI) score. *Baseline is defined as the value at baseline 3 in the confirmatory study. Data are expressed as mean ± standard deviation

The change (mean ± SD) in DLQI score from baseline to each assessment time point was −8.0 ± 4.51 in the switching group and −8.2 ± 4.87 in the extension group at week 4, −9.4 ± 4.63 in the switching group and −10.3 ± 5.10 in the extension group at week 24, and −8.8 ± 4.65 in the switching group and −9.7 ± 5.08 in the extension group at week 52 (Table 2). As shown by time courses of mean change in DLQI score at each assessment time point in Figure 4, the DLQI score remained almost the same throughout the long‐term study.

##### (vi) Proportion of patients with an improvement of 1.5 or more in HDSM‐Ax score

The proportion of patients with an improvement of 1.5 or more in HDSM‐Ax score from baseline to week 52 was 69.1% (65/94 patients) in the switching group and 62.6% (57/91 patients) in the extension group (Table 2).

##### (vii) Change in HDSM‐Ax score

**FIGURE 5:**
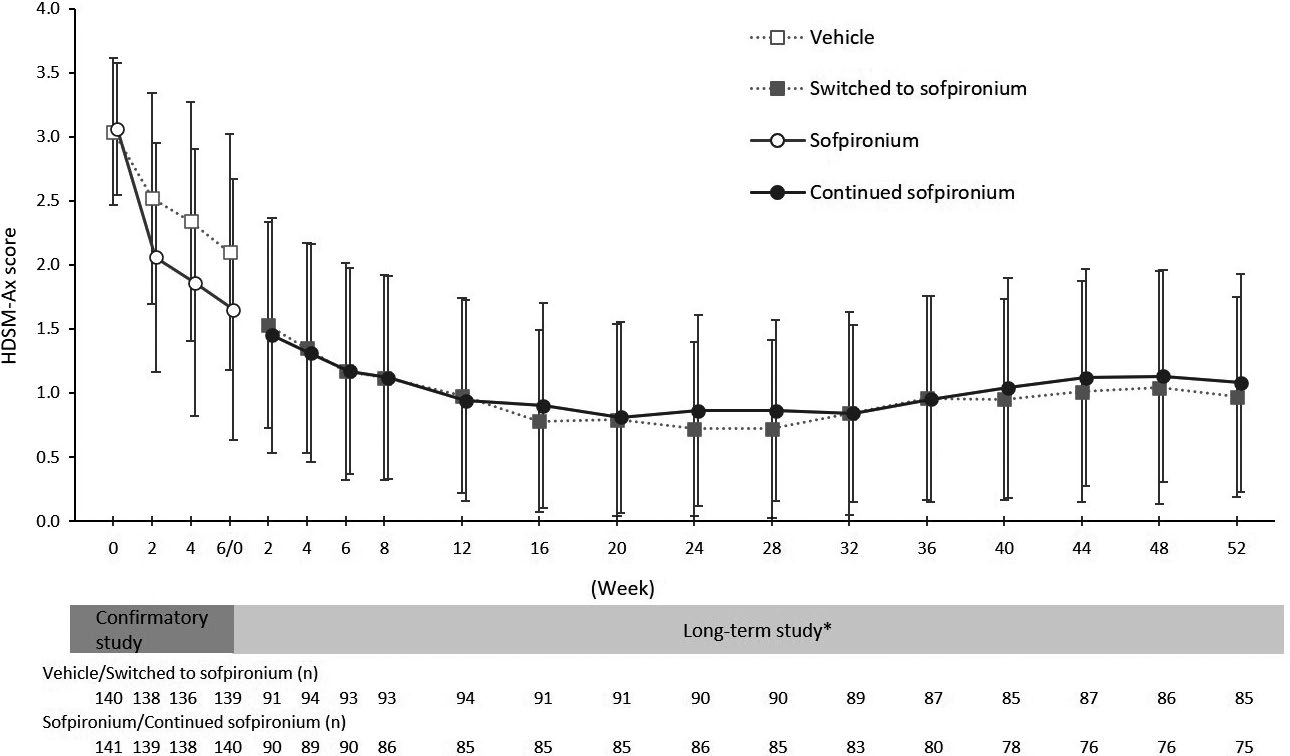
Change in Hyperhidrosis Disease Severity Measure – Axillary (HDSM‐Ax) score. *Baseline is defined as the value at baseline 3 in the confirmatory study. Data are expressed as mean ± standard deviation

The change (mean ± SD) in HDSM‐Ax score from baseline to each assessment time point was −1.56 ± 0.887 in the switching group and −1.63 ± 0.881 in the extension group at week 2, −1.93 ± 0.977 in the switching group and −1.92 ± 0.812 in the extension group at week 6, −2.38 ± 0.765 in the switching group and −2.26 ± 0.802 in the extension group at week 24, and −2.13 ± 0.852 in the switching group and −2.07 ± 0.833 in the extension group at week 52 (Table 2). As shown by time courses of mean change in HDSM‐Ax score at each assessment time point in Figure 5, the HDSM‐Ax score decreased from the baseline to week 6 and remained almost the same thereafter until week 52.

#### 3.5.2 Subgroup analysis of efficacy

##### Analysis by sofpironium treatment status

The proportion of patients with a HDSS of 1 or 2 at week 52 and a 50% or more reduction in total gravimetric weight of sweat from baseline to week 52 was 56.0% (47/84 patients) in those who applied all doses of sofpironium at bedtime and 59.4% (60/101 patients) in those who applied at least one dose of sofpironium at a time other than bedtime.

##### Analysis by concomitant medication/therapy status

The proportion of patients with a HDSS of 1 or 2 at week 52 and a 50% or more reduction in total gravimetric weight of sweat from baseline to week 52 was analyzed separately in patients who received concomitant medication/therapy when the evaluation at week 52 might be affected and those who did not. The proportion of patients who achieved the end‐point was 0% (0/3 patients) in patients who received systemic or topical anticholinergics concomitantly and 58.8% (107/182 patients) in patients who did not; 100% (1/1 patient) in patients who received oral cholinergic agonists, serotonin agonists, β‐blockers, α‐adrenergic agonists, dopamine partial agonists, or tricyclic antidepressants and 57.6% (106/184 patients) in patients who did not; 80.0% (4/5 patients) in patients who received aluminum chloride or medications for hyperhidrosis approved outside Japan and 57.2% (103/180 patients) in patients who did not; 100% (4/4 patients) in patients who received botulinum toxin (axillary administration) and 56.9% (103/181 patients) in patients who did not; 40.0% (2/5 patients) in patients who received styptic‐containing, antiperspirant OTC medications, quasi‐pharmaceutical products, or cosmetics and 58.3% (105/180 patients) in patients who did not; and 0% (0/1 patient) in patients who received axillary laser therapy, surgical therapy, or thoracic sympathectomy and 58.2% (107/184 patients) in patients who did not.

### 3.6 Safety

In this study, the safety was evaluated in the 185 patients of the SAF. The incidence of AEs was 80.9% (76/94 patients) in the switching group and 83.5% (76/91 patients) in the extension group, and the incidence of ADRs was 39.4% (37/94 patients) in the switching group and 45.1% (41/91 patients) in the extension group. By severity, most of the AEs were mild in both groups, with few moderate AEs and no severe AEs (Table 3). Among the common AEs (incidence ≥5% in any treatment group) listed in Table 3, the following occurred in at least 20% of patients in both treatment groups: application site dermatitis in 25.5% (24/94 patients) in the switching group and 33.0% (30/91 patients) in the extension group, and nasopharyngitis in 31.9% (30/94 patients) in the switching group and 23.1% (21/91 patients) in the extension group. Among common ADRs (incidence ≥2% in any treatment group) listed in Table 3, the following occurred in at least 5% of patients in both treatment groups: application site dermatitis in 25.5% (24/94 patients) in the switching group and 29.7% (27/91 patients) in the extension group, application site eczema in 6.4% (6/94 patients) in the switching group and 7.7% (7/91 patients) in the extension group, and application site erythema in 6.4% (6/94 patients) in the switching group and 5.5% (5/91 patients) in the extension group.

**TABLE 3:** Incidence of AEs (SAF)

	Switching group (n = 94)	Extension group (n = 91)
	Incidence, n (%)	
AEs		
Any	76 (80.9%)	76 (83.5%)
Drug‐related AEs (ADRs)	37 (39.4%)	41 (45.1%)
Serious AEs	2 (2.1%)	1 (1.1%)
Discontinuation due to AEs	2 (2.1%)	5 (5.5%)
Death	0	0
AEs by severity		
Mild	71 (75.5%)	69 (75.8%)
Moderate	5 (5.3%)	7 (7.7%)
Severe	0	0
AEs reported in ≥5% of patients		
Application site dermatitis	24 (25.5%)	30 (33.0%)
Nasopharyngitis	30 (31.9%)	21 (23.1%)
Acne	9 (9.6%)	8 (8.8%)
Application site eczema	6 (6.4%)	9 (9.9%)
Eczema	8 (8.5%)	5 (5.5%)
Application site erythema	6 (6.4%)	6 (6.6%)
Dermatitis contact	3 (3.2%)	6 (6.6%)
Influenza	4 (4.3%)	5 (5.5%)
Miliaria	6 (6.4%)	2 (2.2%)
Urticaria	5 (5.3%)	3 (3.3%)
Anticholinergic AEs		
Headache	4 (4.3%)	1 (1.1%)
Mydriasis	1 (1.1%)	2 (2.2%)
Dysuria	1 (1.1%)	1 (1.1%)
Dry mouth	0	1 (1.1%)
Vision blurred	0	1 (1.1%)
Thirst	0	1 (1.1%)
Constipation	1 (1.1%)	0
Insomnia	1 (1.1%)	0
Nausea	0	1 (1.1%)
Drug‐related AEs (ADRs) in ≥2% of patients		
Application site dermatitis	24 (25.5%)	27 (29.7%)
Application site eczema	6 (6.4%)	7 (7.7%)
Application site erythema	6 (6.4%)	5 (5.5%)
Application site pruritus	3 (3.2%)	3 (3.3%)
Mydriasis	1 (1.1%)	2 (2.2%)
Application site dryness	2 (2.1%)	1 (1.1%)
Application site irritation	2 (2.1%)	0

Note

MedDRA ver. 21.1.

Abbreviations: ADRs, adverse drug reactions; AE, adverse event; SAF, safety analysis set.

The incidence rate of AEs at study drug application sites was 38.3% (36/94 patients) in the switching group and 50.5% (46/91 patients) in the extension group, and the incidence rate of ADRs at study drug application site was 38.3% (36/94 patients) in the switching group and 41.8% (38/91 patients) in the extension group. These events were generally mild in severity.

Anticholinergic AEs were headache (four patients in the switching group and one patient in the extension group, respectively), mydriasis (one patient and two patients, respectively), dysuria (one patient each in both groups), dry mouth (one patient in the extension group), vision blurred (one patient in the extension group), thirst (one patient in the extension group), constipation (one patient in the switching group), insomnia (one patient in the switching group), and nausea (one patient in the extension group). Anticholinergic ADR was mydriasis (one patient and two patients, respectively). These events were generally mild in severity and resolved.

In this study, there were no deaths. Other serious AEs occurred in a total of three patients: two patients in the switching group and one patient in the extension group. These serious AEs were prostatic cyst and diverticulitis in one patient each in the switching group, and strabismus in one patient in the extension group. None of these events were considered related to the study drug by the investigator.

Other significant AEs that led to withdrawal from the study were application site eczema (two patients in the extension group), mydriasis (one patient each in both groups), application site dermatitis (one patient in the switching group), vision blurred (one patient in the extension group), glaucoma (one patient in the extension group), and eczema (one patient in the extension group). These AEs were generally mild in severity. The events were resolving or resolved after withdrawal from the study, except for glaucoma, which was lost to follow‐up.

There were no notable changes in vital signs or laboratory values throughout the study. For all variables of local tolerability at week 52, the median score was 0, and the mean score was less than 0.4 in both groups (Table 4).

**TABLE 4:** Variables of local tolerability at week 52

Scores of variables	Switching group (n = 85)		Extension group (n = 75)		Total (n = 160)	
	Mean ± SD	Median	Mean ± SD	Median	Mean ± SD	Median
Physician assessed						
Dryness	0.0 ± 0.19	0.0	0.1 ± 0.38	0.0	0.1 ± 0.30	0.0
Erythema	0.1 ± 0.39	0.0	0.3 ± 0.68	0.0	0.2 ± 0.55	0.0
Scaling	0.0 ± 0.15	0.0	0.1 ± 0.43	0.0	0.1 ± 0.32	0.0
Patient assessed						
Burning	0.1 ± 0.59	0.0	0.1 ± 0.45	0.0	0.1 ± 0.53	0.0
Itching	0.3 ± 0.88	0.0	0.3 ± 0.56	0.0	0.3 ± 0.75	0.0

Abbreviation: SD, standard deviation.

Using the combined data from the preceding confirmatory study and the current long‐term study, the incidence of AEs in each 3‐month interval was analyzed. According to the combined data, the incidence of AEs was 76.6% (180/235 patients) in the entire study period, and the highest incidence by onset time was 53.6% (126/235 patients) in the period from baseline to day 91. According to the combined data, the incidence of ADRs was 40.9% (96/235 patients) in the entire study period, and the highest incidence by onset time was 28.5% (67/235 patients) in the period from baseline to day 91. The incidences of other kinds of AEs did not tend to increase during long‐term treatment (Table 5).

**TABLE 5:** Incidence of AEs by onset time (combined data from the confirmatory and long‐term study)

Incidence, n (%)	Onset time (day)					
	Baseline^a^ to <91 (n = 235)	≥91 to <182 (n = 183)	≥182 to <273 (n = 176)	≥273 to <364 (n = 172)	≥364 (n = 163)	Entire period (n = 235)
AEs	126 (53.6%)	29 (15.8%)	17 (9.7%)	6 (3.5%)	2 (1.2%)	180 (76.6%)
Drug‐related AEs (ADRs)	67 (28.5%)	17 (9.3%)	5 (2.8%)	3 (1.7%)	4 (2.5%)	96 (40.9%)
Death	0	0	0	0	0	0
Serious AEs	0	2 (1.1%)	1 (0.6%)	0	0	3 (1.3%)
Serious drug‐related AEs (ADRs)	0	0	0	0	0	0
Discontinuation due to AEs	5 (2.1%)	1 (0.5%)	1 (0.6%)	1 (0.6%)	0	8 (3.4%)
Discontinuation due to drug‐related AEs (ADRs)	4 (1.7%)	1 (0.5%)	1 (0.6%)	1 (0.6%)	0	7 (3.0%)
AEs at the application site	73 (31.1%)	11 (6.0%)	7 (4.0%)	5 (2.9%)	4 (2.5%)	100 (42.6%)
Drug‐related AEs (ADRs) at the application site	64 (27.2%)	16 (8.7%)	5 (2.8%)	3 (1.7%)	3 (1.8%)	91 (38.7%)
Anticholinergic AEs	5 (2.1%)	8 (4.4%)	3 (1.7%)	0	1 (0.6%)	17 (7.2%)
Anticholinergic drug‐related AEs (ADRs)	4 (1.7%)	3 (1.6%)	0	0	1 (0.6%)	8 (3.4%)

Note

Using the combined data from the preceding confirmatory study and the current long‐term study, the incidence of AEs in each 3‐month interval was analyzed. Multiple episodes of the same AE in the same patient were counted as one patient at the earliest onset time. Multiple episodes of the same AE in the same patient in the same 3‐month interval were counted as one patient.

^a^ Initial day of sofpironium administration.

Abbreviation: ADRs, adverse drug reactions; AE, adverse event.

## 4 DISCUSSION

In this 52‐week long‐term study, a total of 185 Japanese patients with primary axillary hyperhidrosis who completed the preceding confirmatory study of sofpironium (multi‐center, randomized, double‐blind, vehicle‐controlled, parallel‐group study)^10^ started to receive sofpironium (94 patients in the vehicle group and 91 patients in the sofpironium group in the confirmatory study). As many as 161 patients (87.0%) completed the long‐term treatment study at week 52. The high study completion rate clearly shows the efficacy, safety, and tolerability of sofpironium.

The efficacy end‐points in the long‐term study were the same as those in the confirmatory study. In analysis in the FAS, the proportion of patients with a HDSS of 1 or 2 at week 52 and a 50% or more reduction in total gravimetric weight of sweat from baseline to week 52 was 57.4% in the switching group and 58.2% in the extension group. The proportion of patients with a HDSS of 1 or 2 was consistently high from the start of the long‐term study to week 52. In this study, patients for whom data were missing due to, for instance, withdrawal from the study were tabulated as patients who failed to meet the criteria (HDSS was not 1 or 2). This analytical method, in which all patients who could not continue treatment for any reason were handled as treatment failure, is more conservative than the observed case approach. Nonetheless, the efficacy was maintained, showing a high treatment compliance with sofpironium gel formulation. A slight decrease in efficacy after week 24 may be partly explained by withdrawals for personal convenience. In the confirmatory study, the proportion of patients with a HDSS of 1 or 2 at the end of 6‐week treatment and a 50% or more reduction in total gravimetric weight of sweat from baseline to the end of treatment, which was the primary efficacy end‐point, was 53.9% (76/141 patients) in the sofpironium group.^10^ The proportion of patients who achieved this efficacy end‐point at week 52 in the long‐term study was similar to that in the confirmatory study. In addition, all the other efficacy end‐points in the long‐term study supported the finding that the efficacy of sofpironium was maintained until week 52 of treatment.

In subgroup analysis of efficacy, the outcomes in the subgroups of patients treated at bedtime and those treated at a time other than bedtime were similar to each other and to those in the FAS, indicating that time of administration had no effect on the efficacy of sofpironium. In subgroup analysis by concomitant medication/therapy status, sofpironium was effective in the subgroup of patients who concomitantly received aluminum chloride, the first‐line treatment of primary axillary hyperhidrosis in Japan,^3^ and in those who concomitantly received botulinum toxin, the second‐line treatment,^3^ suggesting that these concomitant drugs may have had no effect on the efficacy of sofpironium, although the small sample size precluded an accurate assessment.

In the 52‐week long‐term study, the incidence of AEs was 80.9% (76/94 patients) in the switching group and 83.5% (76/91 patients) in the extension group, and the incidence of ADRs was 39.4% (37/94 patients) in the switching group and 45.1% (41/91 patients) in the extension group. The incidences of AEs and ADRs in the sofpironium group in the confirmatory study were 44.0% (62/141 patients) and 16.3% (23/141 patients), respectively.^10^ In the long‐term study, the following AEs occurred in at least 20% of patients in both treatment groups: application site dermatitis in 25.5% in the switching group and 33.0% in the extension group, and nasopharyngitis in 31.9% in the switching group and 23.1% in the extension group. In the confirmatory study, common AEs (incidence ≥5%) in the sofpironium group were nasopharyngitis in 14.2%, application site dermatitis in 8.5%, and application site erythema in 5.7%.^10^ The most common event was nasopharyngitis, which occurred more frequently in the sofpironium group than in the vehicle group, but all cases of nasopharyngitis in the sofpironium group were incidental cold symptoms and therefore considered unrelated to sofpironium. Application site dermatitis and nasopharyngitis, the common AEs in the long‐term study, were already frequently reported in the 6‐week confirmatory study, although the incidence differed due to a difference in duration of treatment or other reasons. Additionally, favorable local tolerability of sofpironium was shown in the physician assessed dryness, erythema and scaling, and patients assessed burning sensation and itching.

Anticholinergic AEs were headache (four patients in the switching group and one patient in the extension group, respectively), mydriasis (one patient and two patients, respectively), dysuria (one patient in both groups), dry mouth (one patient in the extension group), vision blurred (one patient in the extension group), thirst (one patient in the extension group), constipation (one patient in the switching group), insomnia (one patient in the switching group), and nausea (one patient in the extension group). Anticholinergic ADR was mydriasis (one patient and two patients, respectively). In the 44‐week long‐term study of topical glycopyrronium tosylate (QBREXZA^®^), that is used for the treatment of axillary hyperhidrosis in the USA, the anticholinergic AEs reported among the 550 patient population were dry mouth (93 patients), vision blurred (37 patients), mydriasis (29 patients), urinary hesitation (23 patients), nasal dryness (20 patients), and dry eye (16 patients).^14^ Therefore, the incidence of anticholinergic AEs in this long‐term study of sofpironium was acceptable when compared with that in the long‐term study of topical glycopyrronium tosylate.

In the long‐term study, there were no deaths, and serious AEs occurred in three patients: prostatic cyst, diverticulitis, and strabismus in one patient each. None of these events were considered related to the study drug by the investigator. Almost all patients experienced any AEs, and this seem to be associated with long‐term treatment. Reported AEs were generally mild in severity without any severe AEs.

AEs that led to withdrawal from the study were application site eczema, mydriasis, application site dermatitis, vision blurred, glaucoma, and eczema. These AEs were generally mild in severity and resolving or resolved after withdrawal from the study, except for glaucoma, which was lost to follow‐up. Anticholinergic AEs were headache, mydriasis, dysuria, dry mouth, vision blurred, thirst, constipation, insomnia, and nausea. These events were generally mild in severity and resolved. Since the anticholinergic AEs were generally mild in severity and reversible, the safety risk due to anticholinergic effects of sofpironium is thought to be low and controllable. AEs at the application site of the study drug occurred in 44.3% of patients (82/185 patients) in the two groups and were generally mild in severity. Sofpironium is associated with a risk of dermatitis and irritant reactions at the application site, but this risk is plausible in that sofpironium is intended for topical use and may be controlled by taking appropriate measures such as suspension of treatment at the onset of an event, since these changes were generally mild in severity and did not substantially preclude continued use. There were no notable changes in laboratory values or vital signs. Subgroup analysis of safety by time of administration (at bedtime or not) or concomitant medication/therapy revealed no significant problems (data not shown).

Safety data in the sofpironium group in the confirmatory study and in patients in the long‐term study were combined and tabulated for 3‐month intervals, revealing that the incidence of AEs was highest in the first 3 months, and that the incidences of any events in each interval did not tend to increase during long‐term treatment.

In conclusion, the efficacy was maintained throughout the 52 weeks of treatment. Dermatitis and irritant reactions at the application site were identified as safety risks, but may be controllable. Anticholinergic AEs, possible adverse effects of sofpironium, were generally mild in severity and the incidence was low. No new safety concerns were found in the 52‐week long‐term study. It was shown that sofpironium can be safely used in long‐term treatment for primary axillary hyperhidrosis.

## CONFLICT OF INTEREST

This study was funded by Kaken Pharmaceutical Co., Ltd. H.Y. received a consultancy fee from Kaken Pharmaceutical Co., Ltd. M.A., T.Y., and S. T. are employees of Kaken Pharmaceutical Co., Ltd., and have stock in Kaken Pharmaceutical Co., Ltd. With funding from Kaken Pharmaceutical Co., Ltd., Medical Professional Relations Inc. assisted in the writing and editing of this article.
